# Genome Sequence of a Bovine Rhinitis B Virus Identified in Cattle in Sweden

**DOI:** 10.1128/genomeA.00172-17

**Published:** 2017-05-11

**Authors:** Anne-Lie Blomström, Veslemøy Oma, Mamata Khatri, Hanne H. Hansen, Maria Stokstad, Mikael Berg, Mette Myrmel

**Affiliations:** a Department of Biomedical Sciences and Veterinary Public Health, Section of Virology, Swedish University of Agricultural Sciences, Uppsala, Sweden; b Department of Production Animal Clinical Sciences, Faculty of Veterinary Medicine, Norwegian University of Life Sciences, Oslo, Norway; c Department for Food Safety and Infection Biology, Faculty of Veterinary Medicine, Norwegian University of Life Sciences, Oslo, Norway; d Faculty of Biosciences, Center for Integrative Genetics (CIGENE), Norwegian University of Life Sciences, Ås, Norway

## Abstract

A bovine rhinitis B virus, identified in a calf from Sweden, was genetically characterized. The complete polyprotein was recovered, and phylogenetic analysis showed that this virus has the highest similarity to a bovine rhinitis B virus previously identified in Mexico.

## GENOME ANNOUNCEMENT

Bovine respiratory disease (BRD) is a complex condition that is costly for the cattle industry worldwide. Several viruses have been associated with BRD, such as bovine coronavirus (BCoV), bovine respiratory syncytial virus, bovine parainfluenza virus 3, and bovine herpesvirus type 1 (1, 2). In a sample from an experimental study investigating BCoV (3), a nearly full-length genome of a bovine rhinitis B virus (BRBV) was identified.

A nasal swab was collected from a BcoV-infected calf in Sweden, and the sample was treated with RNase A and DNase prior to RNA extraction. The RNA was reverse transcribed and amplified using the Ovation RNA sequencing (RNA-Seq) system version 2, according to the manufacturer’s instructions (two technical replicates). The two amplified samples were prepared for sequencing using the Illumina Nextera XT DNA library preparation kit, and they were sequenced on the MiSeq platform. Between six and nine million reads were obtained per sample. The raw data from the MiSeq run were quality checked and trimmed (*Q* ≥ 30; maximum number of ambiguities = 2) using CLC Genomics Workbench in order to remove poor data. The reads were mapped to BcoV, and the remaining unmapped reads were *de novo* assembled using CLC Genomics Workbench and subjected to blastx searches using Diamond (4). Apart from BCoV and a low number of phage and retrovirus sequences, another positive-sense single-stranded RNA (ssRNA) virus, BRBV, was recognized. About 9,000 and 11,000 BRBV reads were identified in the 2 data sets. The BRBV reads were extracted and *de novo* creating a contig of 5,760 nucleotides that mapped to the BRBV genome. In order to recover a larger portion of the genome, the contig and all the trimmed raw reads were mapped against both BRBV strain Mexb10 (GenBank accession no. KU159357.1) and TCH5 (GenBank accession no. KU168861.1). This rendered the nearly complete BRBV genome, including the entire sequence coding for the polyprotein (GenBank accession no. KY432299). MEGA6 (5) was used for ClustalW alignment and phylogeny (using the neighbor-joining method and a bootstrap value of 1,000) of the identified BRBV and the other 9 nearly complete BRBV genomes available in GenBank. The BRBV identified in this study (BRBV_SWE1) clustered together with a BRBV strain (Mexb10) collected from beef cattle in Mexico in 2015 (6). The nucleotide sequence similarity between the two sequences was 83.8%. The nucleotide similarity between BRBV_SWE1 and other BRBV strains varied between 71 and 75%. On an amino acid level, the similarity varied between 85 and 94%. Looking at the sequence resemblance across the genome, VP2, VP3, and VP1 had the highest variation. The importance of BRBV as a pathogen in cattle is currently unknown, but BRBV has been identified in several other metagenomic studies investigating BRD (6–8).

### Accession number(s).

The nucleotide sequence of BRBV_SWE1 has been deposited in GenBank under the accession no. KY432299.
